# Versatile formation of supported lipid bilayers from bicellar mixtures of phospholipids and capric acid

**DOI:** 10.1038/s41598-020-70872-8

**Published:** 2020-08-14

**Authors:** Tun Naw Sut, Bo Kyeong Yoon, Soohyun Park, Joshua A. Jackman, Nam-Joon Cho

**Affiliations:** 1 School of Materials Science and Engineering, Nanyang Technological University, 50 Nanyang Avenue, Singapore, 639798 Singapore; 2 School of Chemical Engineering, Sungkyunkwan University, Suwon, 16419 Republic of Korea

**Keywords:** Biosensors, Fatty acids, Phospholipids, Organic molecules in materials science, Self-assembly, Membrane biophysics, Nanobiotechnology

## Abstract

Originally developed for the structural biology field, lipid bicelle nanostructures composed of long- and short-chain phospholipid molecules have emerged as a useful interfacial science tool to fabricate two-dimensional supported lipid bilayers (SLBs) on hydrophilic surfaces due to ease of sample preparation, scalability, and versatility. To improve SLB fabrication prospects, there has been recent interest in replacing the synthetic, short-chain phospholipid component of bicellar mixtures with naturally abundant fatty acids and monoglycerides, i.e., lauric acid and monocaprin. Such options have proven successful under specific conditions, however, there is room for devising more versatile fabrication options, especially in terms of overcoming lipid concentration-dependent SLB formation limitations. Herein, we investigated SLB fabrication by using bicellar mixtures consisting of long-chain phospholipid and capric acid, the latter of which has similar headgroup and chain length properties to lauric acid and monocaprin, respectively. Quartz crystal microbalance-dissipation, epifluorescence microscopy, and fluorescence recovery after photobleaching experiments were conducted to characterize lipid concentration-dependent bicelle adsorption onto silicon dioxide surfaces. We identified that uniform-phase SLB formation occurred independently of total lipid concentration when the ratio of long-chain phospholipid to capric acid molecules (“q-ratio”) was 0.25 or 2.5, which is superior to past results with lauric acid- and monocaprin-containing bicelles in which cases lipid concentration-dependent behavior was observed. Together, these findings demonstrate that capric acid-containing bicelles are versatile tools for SLB fabrication and highlight how the molecular structure of bicelle components can be rationally finetuned to modulate self-assembly processes at solid–liquid interfaces.

## Introduction

Bicelles are an important class of membrane-mimicking lipid nanostructures that self-assemble from mixtures of long- and short-chain phospholipids under appropriate processing conditions^1,2^. Also known as lipid nanodisks^3^ or bilayered mixed micelles^4,5^, bicelles can exist in a wide range of morphologies (e.g., perforated sheets and wormlike micelles) depending on parameters such as temperature, q-ratio (long- to short-chain phospholipid molar ratio), total lipid concentration, and lipid composition, and are widely conceptualized as two-dimensional disks whereby long-chain phospholipids constitute a planar lipid bilayer surface and the short-chain phospholipids form a rimmed edge around the bilayer^6–12^. Since they can exhibit magnetic alignment in some cases, bicellar disks have long been used in the nuclear magnetic resonance spectroscopy field as membrane protein hosts^4,13–16^.

In the interfacial science field, bicelles have also proven to be useful as a lipid nanostructure to fabricate supported lipid bilayers (SLBs), which are extensively used in applications such as biosensors and micropatterned arrays^17–25^. Indeed, bicelle adsorption onto hydrophilic surfaces such as silicon dioxide can initiate a surface-mediated molecular assembly process that yields high-quality SLBs composed of long-chain phospholipid^26,27^. Zeineldin et al*.* first reported SLB formation from bicelles^28^ and there has been extensive efforts to unravel mechanistic aspects of bicelle-mediated SLB formation^29–32^. Within this scope, our group and others have performed systematic investigations to understand the effects of bicelle design parameters, such as q-ratio, total lipid concentration, and sample preparation^33^ as well as other properties such as bicelle surface charge and material properties of the surface^34^ along with ionic strength^35^. Furthermore, it is possible to incorporate cholesterol into bicelles and fabricate cholesterol-enriched SLBs containing at least 30 mol% cholesterol^36^. Altogether, these characterization studies have demonstrated that bicelles offer a versatile approach to SLB fabrication and have advantages over conventional approaches using lipid vesicles, such as easier sample processing, more flexibility of specific size properties of the lipid nanostructure, and lower lipid usage in terms of bulk lipid concentration^37,38^.

To date, bicelles used for SLB fabrication have been made with various long-chain phospholipids ranging from those with high^29^ to low^33^ phase-transition temperatures, however, there has been narrower investigation of the short-chain phospholipid component or substitutes thereof. In particular, 1,2-dihexanoyl-*sn*-glycero-3-phosphocholine (DHPC_6_)^29–32^ and 1,2-diheptanoyl-*sn*-glycero-3-phosphocholine (DHPC_7_)^28,30^ are the two main short-chain phospholipids that have been examined in most SLB fabrication-related studies despite the practical need to find natural substitutes with high abundance. Since it has been possible to form solution-phase bicelles by replacing short-chain phospholipids with detergents and detergent-like molecules^39–42^, we recently demonstrated the ability to fabricate SLBs using bicellar mixtures composed of long-chain 1,2-dioleoyl-*sn*-glycero-3-phosphocholine (DOPC) lipid and detergent-like molecules consistent of a saturated fatty acid called lauric acid (LA) with a 12-carbon long chain^43^ or a saturated monoglyceride called monocaprin (MC) with a 10-carbon long chain^44^. At a q-ratio of 2.5, DOPC/LA bicelles were able to form SLBs across a wide range of lipid concentrations, however, SLB formation was less successful at a q-ratio of 0.25 and only worked at very low lipid concentrations^43^. On the other hand, DOPC/MC bicelles were able to form uniform-phase SLBs across a wider range of q-ratios, albeit with stricter lipid concentration-dependent requirements and other biophysical effects^44^. Indeed, DOPC/MC bicelles yielded SLBs only in an intermediate range of lipid concentrations at a q-ratio of 2.5 while SLB formation was also possible across tested lipid concentrations at a q-ratio of 0.25. Notably, there was also SLB formation in some conditions at a q-ratio of 0.05, however, the SLBs appeared to have phase-separated regions or voids due to nonionic MC molecules. Together, these studies demonstrated that bicellar mixtures consisting of long-chain phospholipids and either fatty acids or monoglycerides are useful for SLB formation, while there is room to improve the molecular design for better fabrication performance.

**Figure 1:**
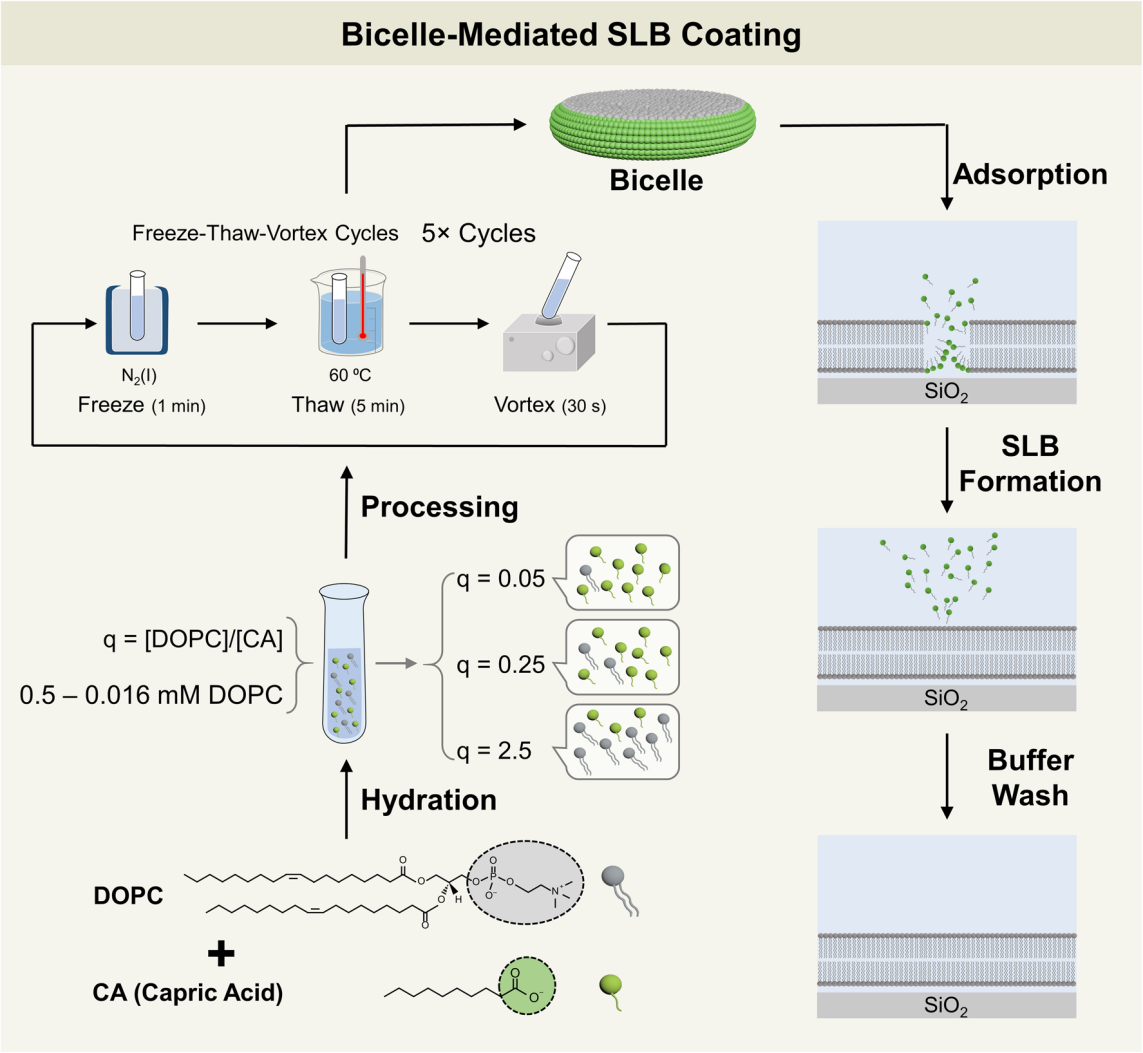
SLB fabrication strategy using DOPC/CA bicellar mixtures. An overview of bicelle sample preparation and SLB formation protocol on silicon dioxide surfaces is presented. The schematic illustration was prepared using the Adobe Photoshop software program (version CS 6, www.adobe.com).

Based on the aforementioned results, we reasoned that the headgroup properties of LA would be well-suited to form uniform-phase SLBs on account of the anionic carboxylic acid functional group while the hydrocarbon chain properties of MC would be useful to form SLBs across a wider range of q-ratio conditions. In particular, DOPC/MC bicelles at the q-ratio of 0.25 were effective to form SLBs across all tested lipid concentrations, which led us to reason that using a fatty acid with equivalent chain length to that of MC would increase the range of effective lipid concentrations for SLB fabrication beyond that observed in the DOPC/LA bicelle case. In addition, since there are more surfactant-like fatty acids than DOPC phospholipids at the q-ratio of 0.25, the fatty acids would likely enhance bicelle deformation via membrane softening and hence promote stronger bicelle-substrate interactions^43^, as opposed to in the high q-ratio regime where DOPC phospholipids are the predominant species. Accordingly, these molecular properties prompted us to investigate the SLB formation potential of bicellar mixtures composed of long-chain phospholipid and capric acid (CA), which is a 10-carbon long, saturated fatty acid which has similar headgroup and chain properties to LA and MC, respectively. Thus, herein, we explored the potential to form SLBs using DOPC/CA bicellar mixtures and systematically investigated the effects of q-ratio and total lipid concentration. The SLB fabrication strategy, including bicelle preparation steps and experimental protocol, is outlined in Fig. 1. The real-time adsorption kinetics of DOPC/CA bicelles onto silicon oxide surfaces were measured by the quartz crystal microbalance-dissipation (QCM-D) and time-lapse fluorescence microscopy techniques. We also conducted fluorescence recovery after photobleaching (FRAP) experiments to quantitatively measure the degree of lateral lipid diffusion in lipid adlayers resulting from bicelle adsorption. The experimental results indicated that DOPC/CA bicelles are able to form uniform-phase SLBs across all tested lipid concentrations at q-ratio values of 0.25 and 2.5, which is a significant improvement compared to DOPC/LA and DOPC/MC bicelles and demonstrates the practical suitability of DOPC/CA bicelles for versatile SLB fabrication.

## Results and discussion

### Quartz crystal microbalance-dissipation

The real-time adsorption kinetics of DOPC/CA bicelles onto silicon dioxide surfaces were measured by the QCM-D technique. The lipid adsorption processes were tracked by measuring changes in the resonance frequency (Δf) and energy dissipation (ΔD) signals of a silicon dioxide-coated piezoelectric quartz sensor chip as a function of elapsed time during the adsorption step. The Δf and ΔD shifts are sensitive to the mass and viscoelastic properties of the adsorbed lipid layer, respectively^45^. Typically, the Δf shift is interpreted to be inversely proportional to the acoustic mass of the adsorbed lipid layer such that a negative Δf shift corresponds to mass adsorption. Likewise, the ΔD shift is related to the viscoelasticity of the adsorbate, whereby a relatively small ΔD shift corresponds to a rigid adlayer like an SLB and a relatively large ΔD shift corresponds to an adsorbed lipid film with a large fraction of hydrodynamically coupled solvent (e.g., adsorbed, intact bicelles or vesicles).

**Figure 2:**
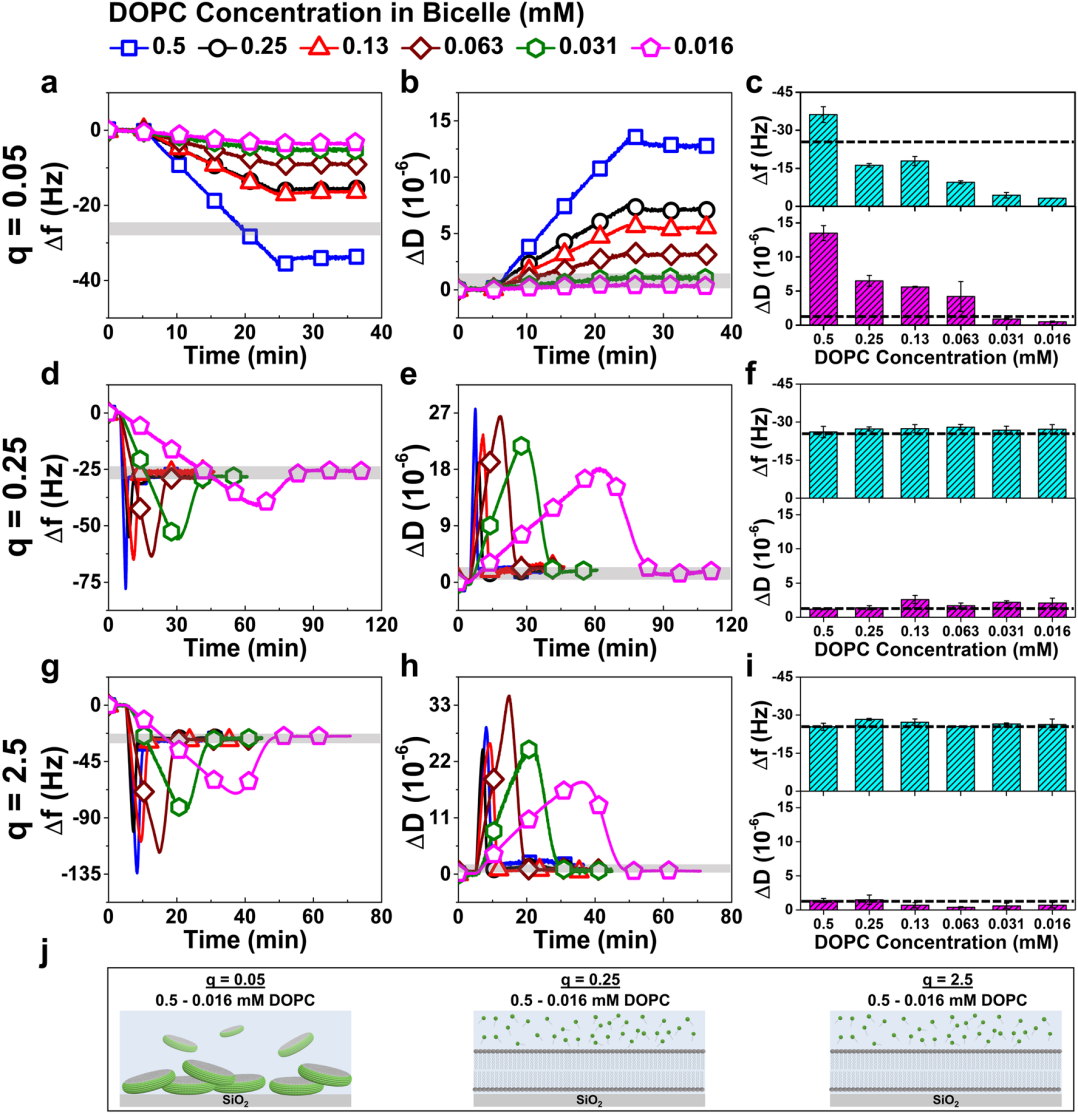
QCM-D measurement results for DOPC/CA bicelle adsorption onto silicon dioxide surfaces. The time-resolved QCM-D (**a**) resonance frequency shifts (Δf) and (**b**) energy dissipation shifts (ΔD) for lipid concentration-dependent adsorption of bicelles at q-ratio of 0.05. Each kinetic curve is plotted from the data of a single representative measurement. The data were collected at a time resolution of 1 Hz and selected data points are overlaid with symbols for distinction of different tested lipid concentrations. (**c**) Column graph reporting the final Δf and ΔD shifts in terms of mean ± standard deviation from *n* = 3 experiments. The corresponding data are presented for the adsorption of bicelles at (**d**–**f**) q-ratio of 0.25 and (**g**–**i**) q-ratio of 2.5. The shaded gray overlays in the time-resolved QCM-D graphs and the dashed lines in the column graphs indicate the typical values for SLBs. (**j**) Graphical illustration of different adsorption scenarios depending on the q-ratio (not drawn to scale; prepared using Adobe Photoshop, version CS 6, www.adobe.com).

In the QCM-D experiments, we systematically investigated the effects of two bicelle parameters: (1) q-ratio values in the range of q = 0.05, 0.25, and 2.5; and (2) total lipid concentrations defined by DOPC lipid concentration in the range of 0.5–0.016 mM DOPC in a two-fold dilution series format. Experimentally, the QCM-D protocol included the following steps: (1) establish a stable baseline signal in buffer for 5 min; (2) inject bicelles under continuous flow; and (3) wash with buffer for at least 10 min. For each q-ratio, the injection time in step (2) was defined based on the initial results obtained at the highest tested lipid concentration (0.5 mM DOPC); the time was 15 min in cases when only monotonic adsorption without rupture occurred (our previous studies indicated that bicelle rupture typically commenced within at least 15 min in cases of SLB formation^43,44^) and as long as necessary for the signals to stabilize in cases of SLB formation. The exact injection time in the latter case depended on the total lipid concentration in the particular experiment because the rate of bicelle adsorption onto the silicon dioxide surface is diffusion-limited and the corresponding diffusion flux is inversely proportional to the total lipid concentration. Afterwards, step (3) was performed in all cases to remove weakly adsorbed lipid molecules. Overall, the lipid adlayer properties were judged by the final Δf and ΔD shifts after step (3) was completed and SLB formation was determined when the final Δf and ΔD shifts were around − 26 Hz and less than 1 × 10^–6^, respectively^45^. The QCM-D measurement results are presented in Fig. 2 and discussed below.

#### *q* = *0.05*

Figure 2a,b present the lipid concentration-dependent QCM-D Δf and ΔD shifts for DOPC/CA bicelle adsorption at q = 0.05. In this condition, there was 20-times more CA than DOPC molecules in the bicellar mixture. At 0.5 mM DOPC, a monotonic adsorption profile was observed and the final Δf and ΔD shifts were around − 36.2 ± 3.1 Hz and 13.5 ± 1.1 × 10^–6^, respectively. Monotonic adsorption was also observed at 0.25 – 0.063 mM DOPC. The final shifts at 0.25 and 0.13 mM DOPC were around half of those at 0.5 mM DOPC, i.e., Δf ~ -17 Hz and ΔD ~ 6.5 × 10^–6^. At 0.063 mM DOPC, the final Δf shift was smaller with values around − 9.5 ± 0.6 Hz and the corresponding ΔD shift was around 4.2 ± 2.2 × 10^–6^. Within this concentration range, the adsorption rate appeared to depend on the total lipid concentration and did not reach saturation within the measurement time span. Together, these findings support that the adsorbed lipid layers were likely composed of intact bicellar disks since the bicelles at this q-ratio are usually discoidal in shape, as has been reported for DOPC/DHPC bicelles^46^ and also for bicelles containing long-chain phospholipids with higher phase transition temperatures^47^ at q ≤ 0.5. By contrast, adsorption uptake was minimal at 0.031 mM DOPC (Δf and ΔD shifts around − 4.4 ± 1.1 Hz and 0.9 ± 0.1 × 10^–6^, respectively) and at 0.016 mM DOPC (Δf and ΔD shifts around − 3.2 ± 0.0 Hz and 0.5 ± 0.1 × 10^–6^, respectively). Overall, DOPC/CA bicelle adsorption generally decreased with decreasing concentration (Fig. 2c). Taken together, the results show that DOPC/CA bicelles at q = 0.05 adsorb but cannot form SLBs.

#### *q* = *0.25*

Figure 2d,e present the lipid concentration-dependent QCM-D Δf and ΔD shifts for DOPC/CA bicelle adsorption at q = 0.25. In this condition, there was 4-times more CA than DOPC molecules in the bicellar mixture. A two-step adsorption profile was observed at all tested lipid concentrations, indicating that bicelles initially adsorb and remain intact until there is a sufficiently high surface coverage of adsorbed bicelles to spontaneously trigger fusion. As a result, the lipid components in the bicellar mixture undergo a structural transformation whereby DOPC phospholipids self-assemble to form an SLB on the silicon dioxide surface and CA molecules return to the bulk solution^33^. In line with the expected diffusion-limited adsorption of the DOPC/CA bicelles, the elapsed time until the critical coverage of adsorbed bicelles and accordingly the time scale of the SLB formation process was greater at lower lipid concentrations. In all cases, the final Δf and ΔD shifts were around − 25 to − 29 Hz and 1 to 3 × 10^–6^, respectively. The QCM-D adsorption kinetics and final measurement responses are generally within the typical range for an SLB. Notably, however, the ΔD shifts are relatively higher than usually seen for complete SLBs and suggest that some intact bicelles remain adsorbed on the silicon dioxide, likely in the form of bicellar disks at this q-ratio^46–48^. Collectively, the QCM-D measurement results show that DOPC/CA bicelles at q = 0.25 can adsorb and spontaneously rupture at all tested lipid concentrations to yield SLBs while the final values indicated the likely presence of some intact bicelles (Fig. 2f).

#### *q* = *2.5*

Figure 2g,h present the lipid concentration-dependent QCM-D Δf and ΔD shifts for DOPC/CA bicelle adsorption at q = 2.5. In this condition, there was 2.5-times more DOPC than CA molecules in the bicellar mixture. As with bicelle adsorption at q = 0.25, two-step adsorption profiles occurred for bicelles at q = 2.5 across all tested lipid concentrations while the corresponding kinetics were quicker in general. Overall, the QCM-D shifts indicated the SLB formation, with final Δf and ΔD values around − 25 to − 28 Hz and 0.4 to 1.5 × 10^–6^, respectively. At this q-ratio, SLB formation likely involved the adsorption and spontaneous rupture of spherical bicelles rather than discoidal bicelles as at q = 0.05 and 0.25, since DOPC/DHPC bicelles^49^ and related ones^50^ have been shown to possess a spherical shape at similar q-ratios. Thus, the QCM-D data support that DOPC/CA bicelles at q = 2.5 can form SLBs at all tested concentrations while optimal conditions corresponded to 0.13–0.016 mM DOPC on account of low ΔD values that are acceptable for good-quality SLBs based on generally practiced standards used in the field, i.e., around or less than 1 × 10^–6^ (Fig. 2i).

In summary, DOPC/CA bicelle adsorption onto silicon dioxide surfaces resulted in the following outcomes depending on the q-ratio and occurred largely independent of the total lipid concentration: intact bicelle adsorption at q = 0.05; SLB formation with some unruptured bicelles at q = 0.25; and good-quality SLB formation at q = 2.5 (Fig. 2j).

### Time-lapse fluorescence microscopy

**Figure 3:**
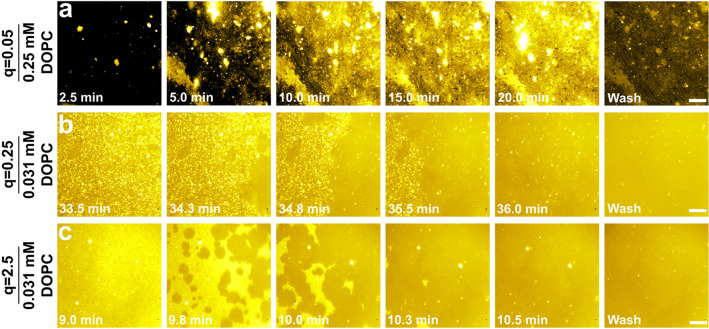
Fluorescence microscopy imaging of DOPC/CA bicelle adsorption at different q-ratio values. The measurements were conducted using fluorescently labeled bicelles with the following parameters: (**a**) q-ratio of 0.05 and 0.25 mM DOPC; (**b**) q-ratio of 0.25 and 0.031 mM DOPC; and (**c**) q-ratio of 2.5 and 0.031 mM DOPC. All scale bars are 20 μm.

Time-lapse fluorescence microscopy experiments were also conducted to characterize DOPC/CA bicelle adsorption for selected cases based on the QCM-D results. In these experiments, the long-chain phospholipid component in the bicellar mixture consisted of a 99.5/0.5 mol% mixture of DOPC and a fluorescently labeled, long-chain phospholipid analogue. A representative case was tested for each q-ratio and described as follows: intact bicelle adsorption at q = 0.05 with 0.25 mM DOPC and SLB formation at q = 0.25 and at q = 2.5 with 0.031 mM DOPC. Figure 3 presents the time-lapse micrographs for bicelle adsorption onto hydrophilic glass surfaces within a microfluidic chamber and the initial time point (*t* = 0 min) corresponds to when the injected bicelle solution reached the measurement chamber. For the intact bicelle adsorption case, the first snapshot corresponds to the surface coverage of adsorbed bicelles at that time point and the subsequent snapshots show the corresponding surface coverage at the noted time points. For the SLB formation cases, the first snapshot corresponds to when the critical surface coverage of adsorbed bicelles was reached and immediately prior to the onset of bicelle fusion and subsequent SLB propagation, which are captured in the subsequent snapshots.

At q = 0.05 and 0.25 mM DOPC, there was progressive adsorption of DOPC/CA bicelles over time, as indicated by greater fluorescence intensity (Fig. 3a). However, bicelle fusion was not observed and some bright spots remained on the surface after a buffer washing step was performed to remove weakly adsorbed lipid molecules. These data agree well with the QCM-D results that showed monotonic adsorption without SLB formation.

On the other hand, bicelle adsorption at q = 0.25 and 0.031 mM DOPC was more gradual and it took around 33.5 min until there was a critical coverage of adsorbed bicelles on the surface (Fig. 3b). The fusion process and SLB propagation commenced around 34.3 min and lasted until around 36.0 min. Upon buffer washing, a small number of bright spots remained on the surface, which is likely due to a small fraction of intact bicelles and is consistent with the QCM-D data.

In addition, the adsorption of bicelles at q = 2.5 and 0.031 mM DOPC occurred more quickly than the q = 0.25 case and the time to reach the critical coverage was only around 9.0 min (Fig. 3c). In this case, bicelle fusion and SLB propagation occurred quickly within around 1.5 min. A very small fraction of intact bicelles remained in the SLB upon buffer washing, as indicated by small bright dots although less so than in the q = 0.25 case (see Supplementary Figure 1 for quantitative comparison of the number of bright spots in SLBs formed from DOPC/CA bicelles at the two q-ratios). Together, the fluorescence microscopy data sets agree well with the QCM-D results and support bicelle adsorption and rupture to yield SLBs at q = 0.25 and at q = 2.5.

### Fluorescence recovery after photobleaching

**Figure 4:**
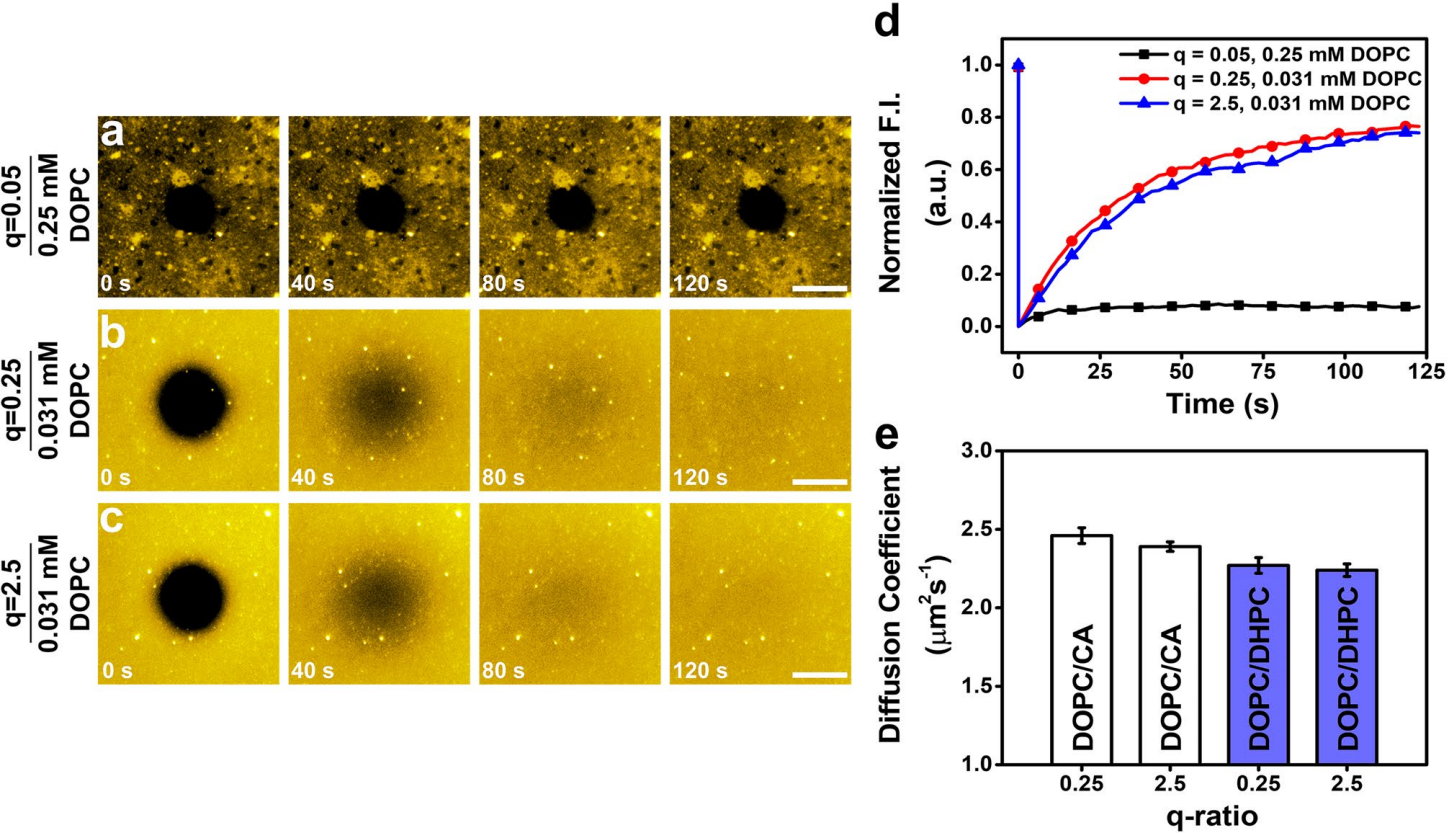
Time-lapse fluorescence recovery profiles and diffusion coefficients of adsorbed lipid layers in FRAP measurements. FRAP measurements were conducted on lipid adlayers formed using bicelles with the following parameters: (**a**) q-ratio of 0.05 and 0.25 mM DOPC; (**b**) q-ratio of 0.25 and 0.031 mM DOPC; and (**c**) q-ratio of 2.5 and 0.031 mM DOPC. All scale bars are 20 μm. (**d**) Time-dependent fluorescence recovery profiles corresponding to data in (**a**–**c**). The fluorescence intensity (F.I.) values were normalized to the value before photobleaching (defined as 1 a.u.). (**e**) Mean diffusion coefficient values are reported from *n* = 6 measurements on different regions of SLBs formed from DOPC/CA bicelles (white columns) at q-ratio values of 0.25 and 2.5 [see (**b**,**c**)] and compared to previously reported values for SLBs formed from DOPC/DHPC bicelles (blue columns; see Ref.^33^). All tested bicelle samples had 0.031 mM DOPC lipid concentration.

In line with the time-lapse fluorescence microscopy experiments, additional FRAP measurements were conducted to quantitatively characterize lateral lipid diffusion within the lipid adlayers, which consisted of either intact bicelles or an SLB depending on the bicelle conditions. In the FRAP experiments, a 20-μm wide spot was photobleached in the lipid adlayer, resulting in the irreversible quenching of fluorescent lipids only within that spot. Then, the gradual recovery of fluorescence intensity within the bleached spot was monitored due to the lateral diffusion between non-fluorescent lipid molecules in the bleached spot and fluorescent molecules in the surrounding regions of the lipid adlayer. The time-dependent recovery profile was then analyzed^51^ in order to determine the diffusion coefficient for laterally diffusing lipid molecules within the adlayer. The time-lapse FRAP micrographs, time-dependent fluorescence recovery profiles, and computed diffusion coefficients for each experimental case are presented in Fig. 4.

For DOPC/CA bicelles at q = 0.05, fluorescence recovery was not observed, indicating the lack of lateral lipid diffusion which is consistent with the adsorbed bicelle layer that forms at this condition (Fig. 4a). This case is analogous to the formation of an intact vesicle layer whereby adsorbed vesicles do not rupture and, as a result, lipid molecules within the adlayer are immobile and there is also no fluorescence recovery^35,52,53^. In marked contrast, the fluorescence intensity within the bleached spot was quickly recovered for SLB adlayers formed using bicelles at q = 0.25 (Fig. 4b) and at q = 2.5 (Fig. 4c). The corresponding time-dependent fluorescence recovery profiles are presented in Fig. 4d. This finding supports that the SLBs formed in these cases exhibit lateral lipid diffusion and the computed diffusion coefficient values were around 2.46 ± 0.05 µm^2^ s^−1^ and 2.39 ± 0.03 µm^2^ s^−1^ for the q = 0.25 and q = 2.5 cases, respectively, which agree well with past literature values reported for SLBs fabricated from DOPC/DHPC bicelles^33^ (Fig. 4e). In addition, the measured diffusion coefficient values are similar to those of SLBs formed from pure DOPC vesicles using the vesicle fusion method, which typically contain a small fraction of unruptured vesicles, as discussed in Ref. 33. While we have noted that there is a low fraction of some intact CA-containing bicelles that remain in the SLBs formed from DOPC/CA bicelles, it is not thermodynamically favorable for relatively short-chain CA molecules to remain in phospholipid SLBs. Residual CA molecules, if present, would return to the bulk solution and be rinsed away during the buffer washing step. For example, it has been observed that treatment of a DOPC SLB with CA monomers has negligible effect on the FRAP-measured diffusion coefficient^54^.

### Comparison of SLB formation conditions

**Figure 5:**
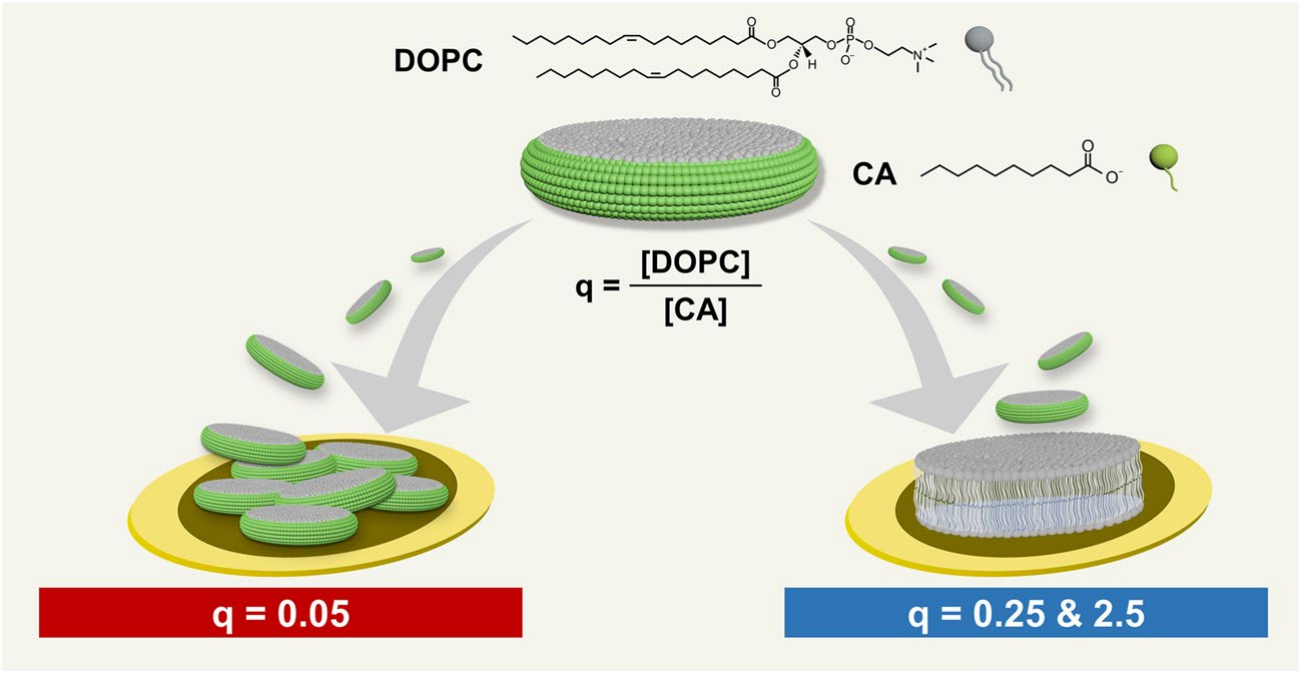
Summary of DOPC/CA bicelle adsorption outcomes depending on the q-ratio parameter. At each tested q-ratio, DOPC/CA bicelle adsorption yielded consistent outcomes, either intact bicelle adsorption or SLB formation, largely independent of total lipid concentration. The illustrative summary was prepared using the Adobe Photoshop software program (version CS 6, www.adobe.com).

The results of the present study demonstrate that uniform-phase fluid SLBs can be fabricated from DOPC/CA bicelles at q-ratios of 0.25 and 2.5 with all tested lipid concentrations in the range of 0.5–0.016 mM DOPC, which suggests that SLB formation occurs largely independently of total lipid concentration. A summary overview of these conditions is provided in Fig. 5. Compared to DOPC/MC bicelles^44^ in which case MC is the monoglyceride derivative of CA, DOPC/CA bicelles yield higher-quality SLBs in terms of uniform-phase properties (homogeneity) for bicelles prepared using identical protocols and with equivalent q-ratio values and lipid concentrations. Indeed, as demonstrated previously, SLBs fabricated using DOPC/MC bicelles sometimes exhibit phase separation, likely due to the ability of MC molecules to intercalate into the bilayer by virtue of hydrogen-bonding capacity with phospholipid headgroups. By contrast, as demonstrated in this work, SLBs fabricated using DOPC/CA bicelles exhibit uniform-phase properties in all successful cases. This difference can be attributed to the ionic nature of CA molecules that limits hydrogen bonding capacity with phospholipids and therefore shortens the residence time in SLBs, which minimizes membrane intercalation and potential membrane-disruptive effects^54^. On the other hand, the SLB conditions of DOPC/CA bicelles are wider-ranging than those of DOPC/LA bicelles^43^ (q = 0.25 with 0.031–0.016 mM DOPC & q = 2.5 with 0.5–0.016 mM DOPC). Nevertheless, the SLBs fabricated from both DOPC/CA and DOPC/LA bicelles have similar phase uniformity owing to similar headgroup properties of the fatty acid molecules (i.e., lack of hydrogen bonding capacity) that limits membrane intercalation and favors return of these molecules to the bulk solution. These findings demonstrate that DOPC/CA bicellar mixtures are a versatile option for bicelle-mediated SLB formation and work at different q-ratios largely independent of total lipid concentration. From a user perspective, it should be noted that higher lipid concentrations lead to quicker SLB fabrication in general while optimal conditions typically involve intermediate lipid concentrations in order to balance fabrication speed with lipid consumption, which is especially important when designing more biomimetic SLB platforms that incorporate precious biological components.

## Conclusion

The findings in this work demonstrate that DOPC/CA bicelles are capable of forming uniform-phase SLBs with fluidic properties at q-ratio values of 0.25 and 2.5. An advantageous feature is that the SLB formation results in these conditions were largely consistent across different tested lipid concentrations. While lipid bicelles have long proven to be promising tools for SLB formation, the recent introduction of natural fatty acids and monoglycerides as replacements for synthetic short-chain phospholipid components in bicellar mixtures opens the door to wider usage. These results further demonstrate the importance of finetuning the molecular structure of bicelle components to achieve target performance outcomes. In this case, we selected the molecular structure of CA to hopefully achieve a balance between the advantageous properties of LA fatty acid and MC monoglyceride, and this approach proved useful for SLB fabrication. The characterization results obtained using various surface-sensitive measurement techniques demonstrated the high SLB fabrication quality and reproducibility that can be achieved with DOPC/CA bicelles. Looking forward, such capabilities might be further extended to design bicellar mixtures with additional biological functionalities or to incorporate other macromolecular components such as membrane proteins, sterols, and signaling lipids into SLB platforms.

## Materials and methods

### Reagents

1,2-Dioleoyl-*sn*-glycero-3-phosphocholine (DOPC) and 1,2-dioleoyl-*sn*-glycero-3-phosphoethanolamine-N-(lissamine rhodamine B sulfonyl) (Rh-PE) lipids were obtained as chloroform stock solutions (Avanti Polar Lipids, Alabaster, AL). Capric acid (CA) and other laboratory reagents were procured from Sigma-Aldrich (St. Louis, MO). All aqueous solutions were prepared using deionized water that had been treated using a Milli-Q purification system (MilliporeSigma, Burlington, MA). The aqueous buffer solution used for all experiments consisted of 10 mM Tris and 150 mM NaCl and the solution pH was fixed at 7.5.

### Bicelle preparation

Bicelles were prepared using the optimized processing protocol, as previously described^43,44^. A chloroform solution of long-chain phospholipids, either 100 mol% DOPC 99.5/0.5 mol% DOPC/Rh-PE lipids depending on the experiment, was placed in a glass vial and the chloroform solvent was evaporated under slow rotation in order to form a dry lipid film on the sidewalls of the vial. After overnight storage in a vacuum desiccator, the lipid sample was hydrated in an aqueous buffer containing 20, 4, or 0.4 mM CA so that the long-chain phospholipid concentration was 1 mM and the final q-ratio of the sample was 0.05, 0.25, or 2.5, respectively. The hydrated lipid samples were then processed into bicelles based on five cycles of the following protocol steps^33^: 1-min flash freezing in liquid nitrogen; 5-min thawing in 60 °C water; and 30-s vortexing. Before experiment, an aliquot of the stock bicelle suspension was diluted in buffer solution to the appropriate test concentration.

### Quartz crystal microbalance-dissipation

QCM-D experiments were conducted to track bicelle adsorption kinetics on silicon dioxide-coated sensor chips by using a Q-Sense E4 instrument (Biolin Scientific AB, Stockholm, Sweden), as previously described^43,44^. Immediately before experiment, the QCM-D sensor chips were repeatedly rinsed with water and ethanol, and then air dried with nitrogen gas before oxygen plasma treatment for 1 min in a vacuum chamber (PDC-002, Harrick Plasma, Ithaca, NY). Liquid samples were introduced into the measurement chambers under continuous flow conditions at a volumetric rate of 50 µL min^−1^, as regulated by a Reglo Digital MS-4/6 peristaltic pump (Ismatec, Glattsburg, Switzerland). Measurement data were collected with a time resolution of 1 Hz at the 3rd–11th odd overtones by the Q-Soft software package (BiolinScientific AB), and the overtone-normalized data from the 5th odd overtone are reported. Data processing was completed using the Q-Tools (Biolin Scientific AB) and OriginPro (OriginLab, Northampton, MA) software programs.

### Epifluorescence microscopy

Time-lapse imaging experiments were conducted, as previously described^43,44^, to track bicelle adsorption and SLB formation on glass surfaces by using an Eclipse Ti-E inverted microscope (Nikon, Tokyo, Japan) with a 60 × oil-immersion objective lens and a numerical aperture of 1.49. An Intensilight C-HGFIE mercury-fiber illuminator (Nikon) was used for light emission, which was passed through a TRITC filter set. Fluorescence micrographs were recorded using an iXon3 897 electron multiplying charge coupled device camera (Andor Technology, Belfast, Northern Ireland) and the recording rate was 1 frame per 3 s. For experiments, an oxygen plasma-treated cover glass substrate (No. 1.5H, ibidi GmbH, Martinsried, Germany) was placed within a sticky-Slide VI 0.4 microfluidic chamber (ibidi GmbH) and liquid samples were introduced under continuous flow conditions at a volumetric rate of 50 µL min^−1^, as regulated by a Reglo Digital MS-4/6 peristaltic pump (Ismatec).

### Fluorescence recovery after photobleaching

FRAP experiments were conducted to measure the extent of lateral lipid diffusion in the lipid adlayer resulting from bicelle adsorption onto glass surfaces, as previously described^43,44^. The lipid adlayers contained 0.5 mol% Rh-PE fluorescent lipid and a spot of 20 μm diameter was photobleached within the lipid adlayer by using a single-mode, 532-nm laser with 100 mW power (Coherent Inc., Santa Clara, CA). Afterwards, time-lapse fluorescence micrographs of the bleached spot were recorded for 2 min at 3-s intervals to track fluorescence signal recovery and the time-dependent recovery profile was analyzed by the Hankel transform method^51^ in order to calculate the corresponding diffusion coefficient.

## Supplementary information

Supplementary Information.
