# Supplementary material for: Versatile formation of supported lipid bilayers from bicellar mixtures of phospholipids and capric acid
Source: Sci Rep. 2020 Aug 14;10:13849. doi: 10.1038/s41598-020-70872-8 (PMC7427796; doi:10.1038/s41598-020-70872-8)
Supplement: Supplementary file 1 — Supplementary Information. [file 41598_2020_70872_MOESM1_ESM.pdf]

Supplementary Information

Versatile Formation of Supported Lipid Bilayers from Bicellar Mixtures of Phospholipids and Capric Acid

Tun Naw Sut[1,2,†], Bo Kyeong Yoon[2,†], Soohyun Park[1], Joshua A. Jackman[*,2], Nam-Joon Cho[*,1]

[1]School of Materials Science and Engineering, Nanyang Technological University, 50 Nanyang Avenue 639798, Singapore
[2]School of Chemical Engineering, Sungkyunkwan University, Suwon 16419, Republic of Korea

[†]These authors contributed equally to this work.

*Address correspondence to:

E-mail: njcho@ntu.edu.sg (N.-J.C.) and jjackman@skku.edu (J.A.J.)

Supplementary Figures

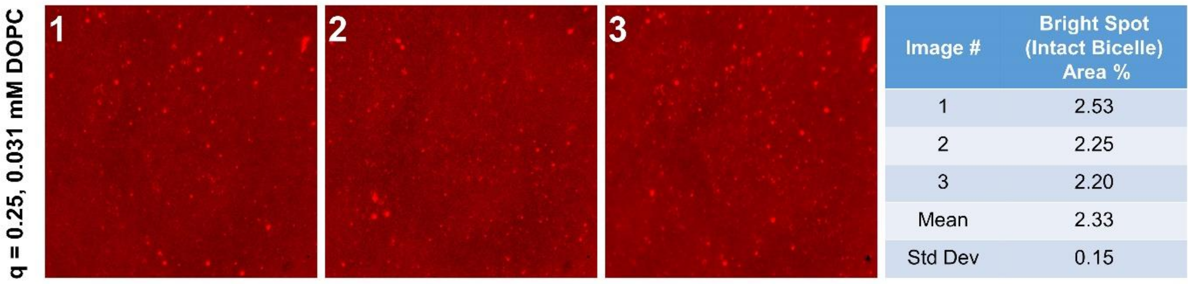

Image #	Bright Spot (Intact Bicelle) Area %
1	2.53
2	2.25
3	2.20
Mean	2.33
Std Dev	0.15

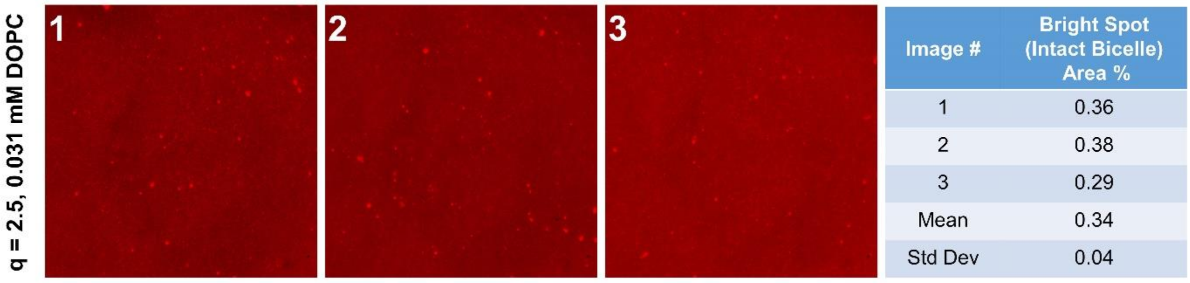

Image #	Bright Spot (Intact Bicelle) Area %
1	0.36
2	0.38
3	0.29
Mean	0.34
Std Dev	0.04

Supplementary Figure 1. Fluorescence microscopy image quantification of bright spots corresponding to intact bicelles present in a DOPC SLB on a glass surface, which had been formed using DOPC/CA bicelles at 0.031 mM DOPC concentration and q-ratios of 0.25 (top row) and 2.5 (bottom row). Image analysis was performed using the ImageJ program (National Institutes of Health, Bethesda, MD) as follows: change the image to a binary image by setting a threshold such that the bright spots (intact bicelles) appear as white and the background (SLB) as black; analyze the binary image by setting the program to count the number of white particles and obtain the area of each white particle; and based on the results, calculate the area percentage of the white particles over the entire area of the image. The mean and standard deviation are reported from *n*=3 technical replicates; each image corresponds to one of the replicates.
